# Supplementary material for: Control of telomere length in yeast by SUMOylated PCNA and the Elg1 PCNA unloader
Source: eLife. 2023 Aug 2;12:RP86990. doi: 10.7554/eLife.86990 (PMC10396338; doi:10.7554/eLife.86990)
Supplement: Figure 1—source data 1. [file elife-86990-fig1-data1.zip › Figure 1B.pptx]

## Slide 1
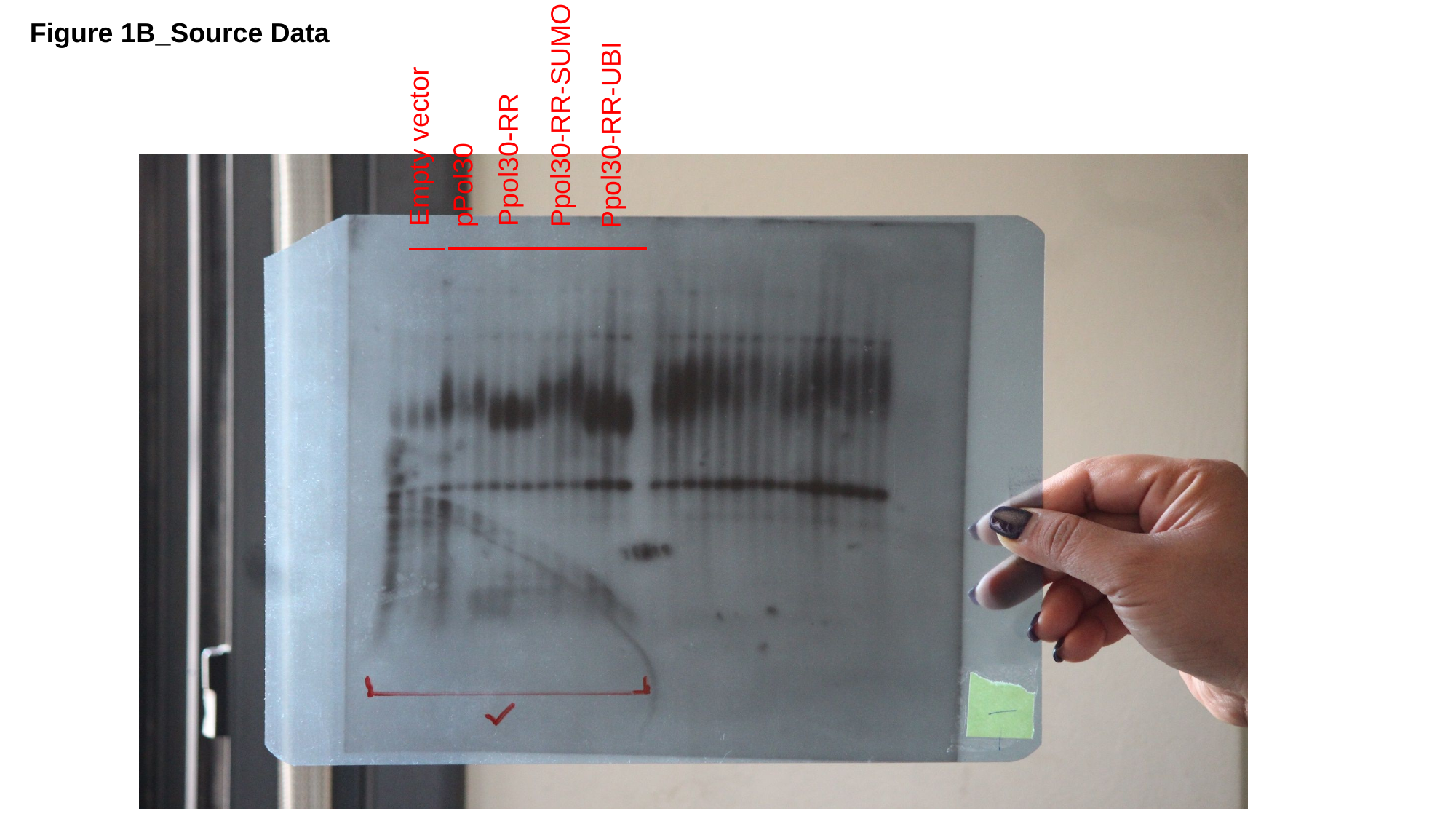

Figure 1B_Source Data
Ppol30-RR-SUMO
Ppol30-RR-UBI
Ppol30-RR
Empty vector
pPol30

## Slide 2
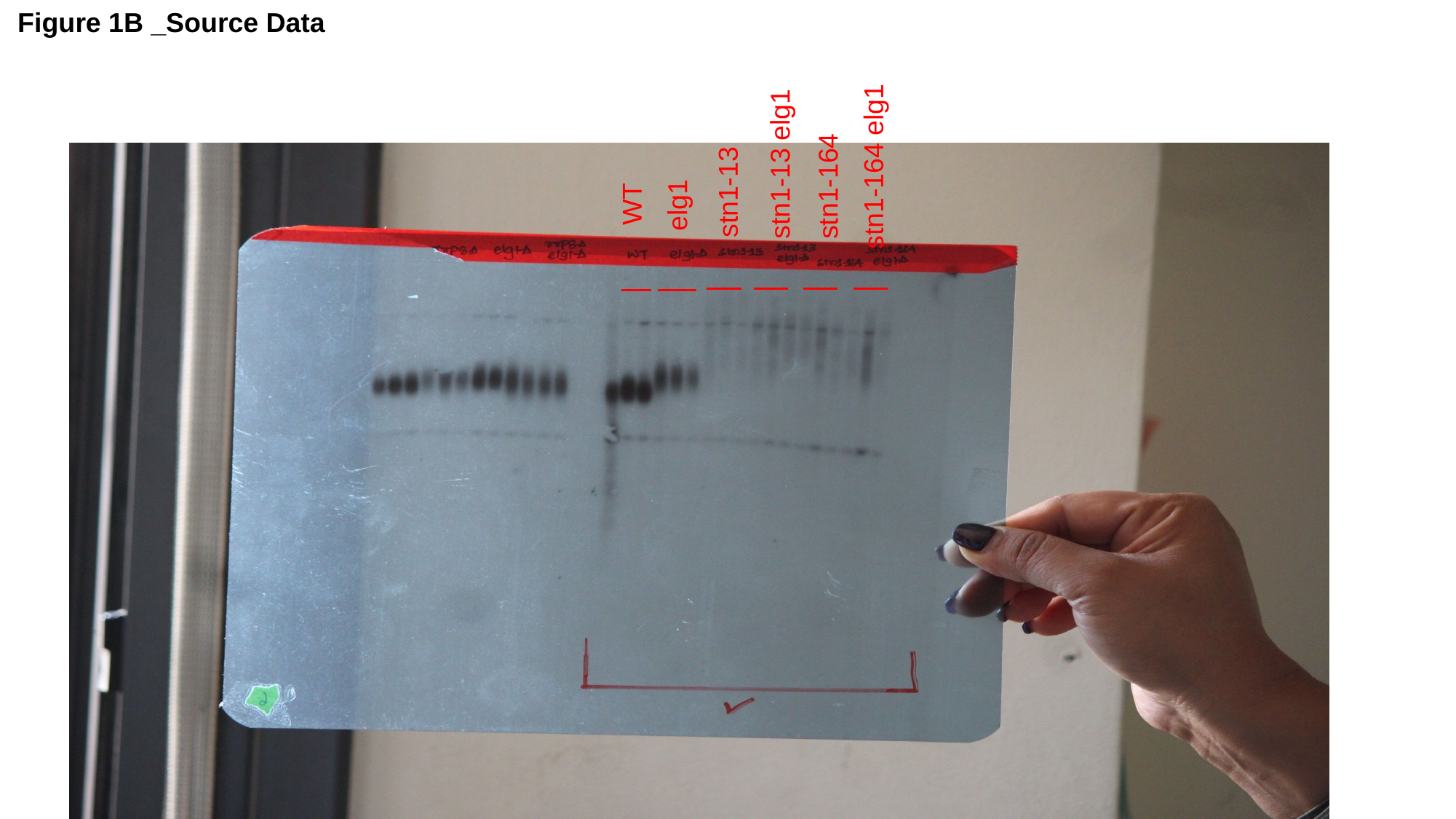

Figure 1B _Source Data
stn1-13 elg1
stn1-164 elg1
stn1-164
stn1-13
WT
elg1
