# Supplementary material for: Control of telomere length in yeast by SUMOylated PCNA and the Elg1 PCNA unloader
Source: eLife. 2023 Aug 2;12:RP86990. doi: 10.7554/eLife.86990 (PMC10396338; doi:10.7554/eLife.86990)
Supplement: Figure 2—source data 1. [file elife-86990-fig2-data1.zip › Figure 2/Fig 2BCenter.pptx]

## Slide 1
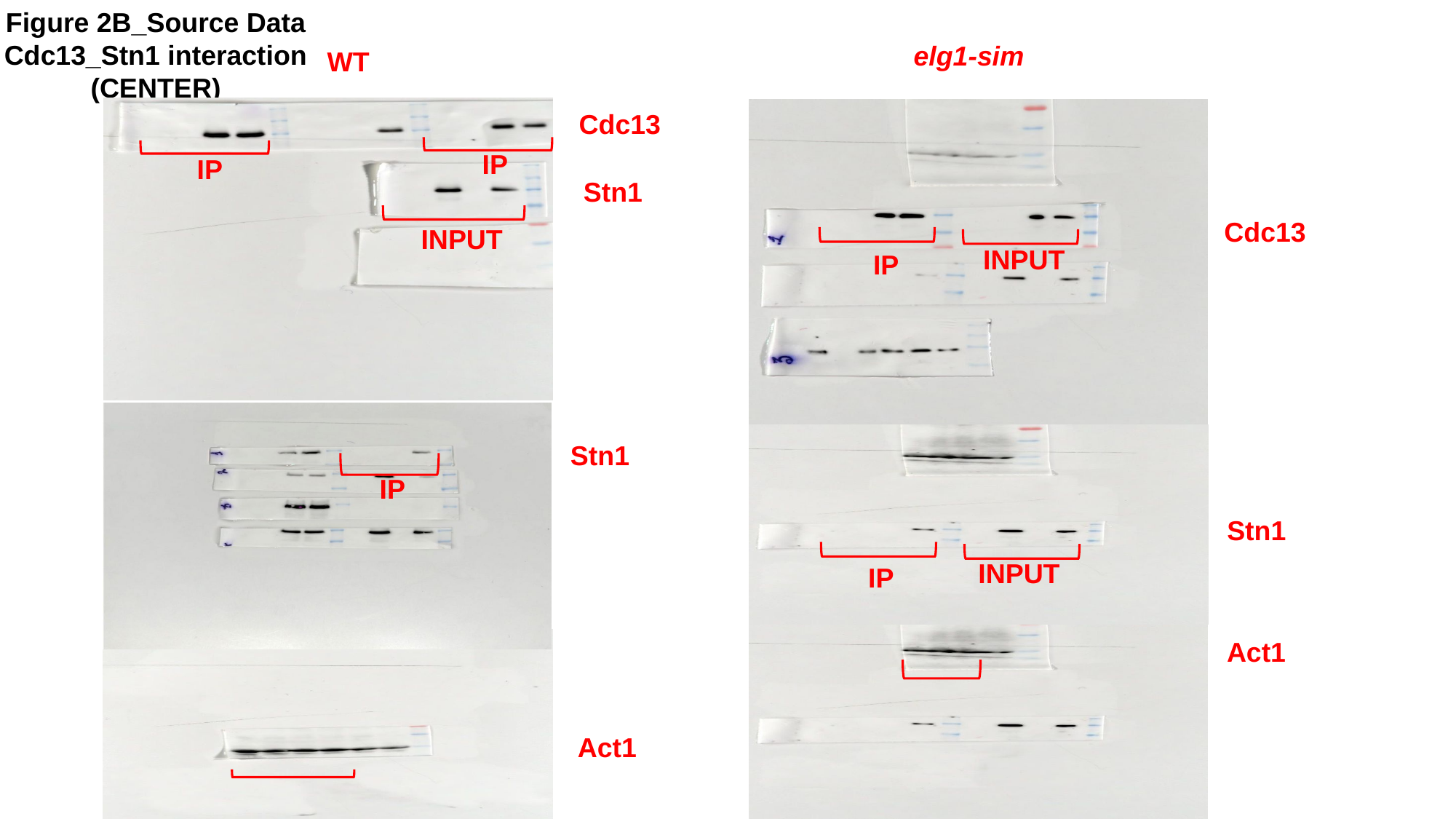

Figure 2B_Source Data
Cdc13_Stn1 interaction
(CENTER)
elg1-sim
WT
Cdc13
IP
IP
Stn1
Cdc13
INPUT
INPUT
IP
Stn1
IP
Stn1
INPUT
IP
Act1
Act1
