# Supplementary material for: Control of telomere length in yeast by SUMOylated PCNA and the Elg1 PCNA unloader
Source: eLife. 2023 Aug 2;12:RP86990. doi: 10.7554/eLife.86990 (PMC10396338; doi:10.7554/eLife.86990)
Supplement: Figure 2—source data 1. [file elife-86990-fig2-data1.zip › Figure 2/Fig 2 B with MWM(1).pptx]

## Slide 1
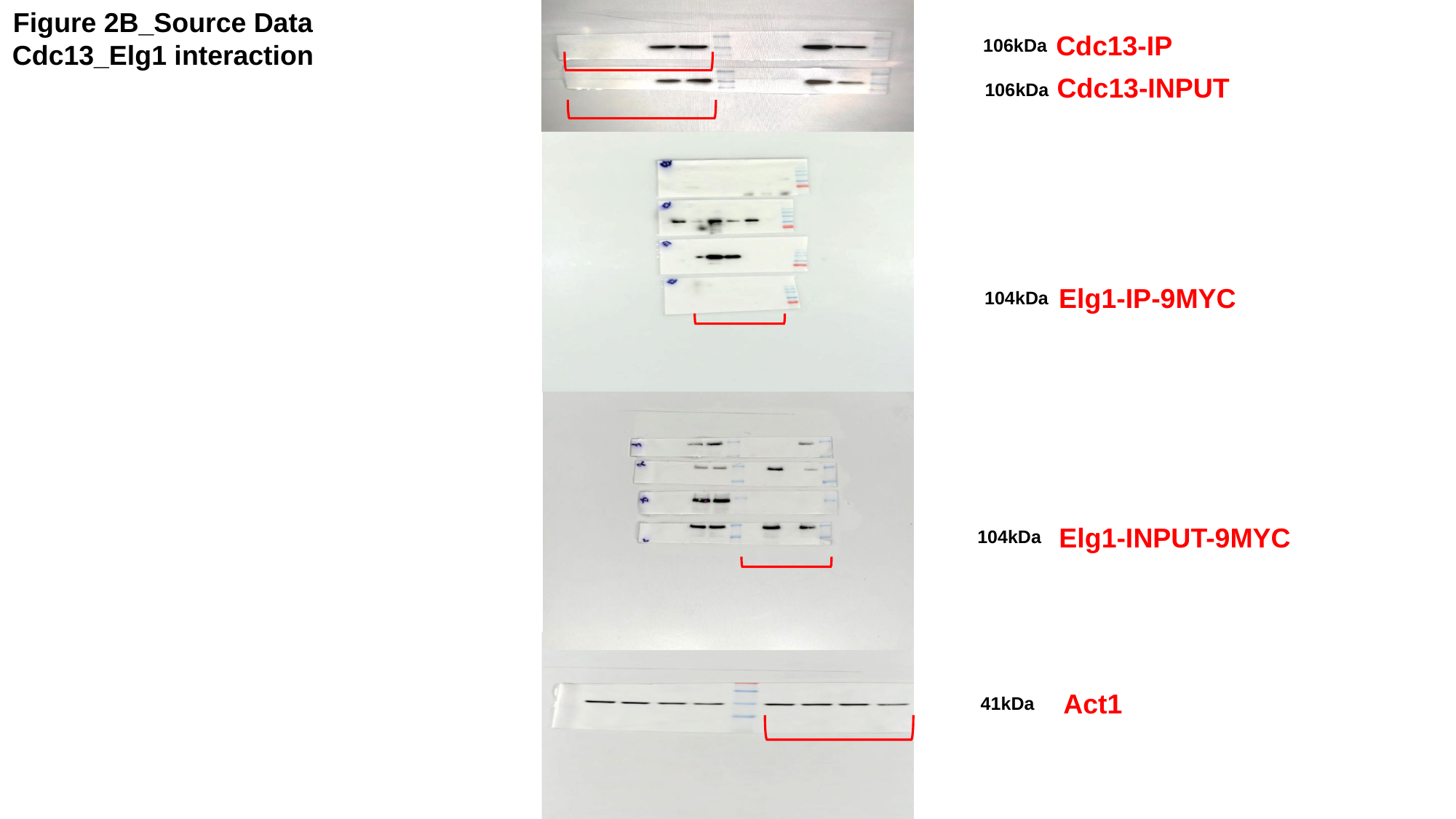

Figure 2B_Source Data
Cdc13_Elg1 interaction
Cdc13-IP
106kDa
Cdc13-INPUT
106kDa
Elg1-IP-9MYC
104kDa
Elg1-INPUT-9MYC
104kDa
Act1
41kDa

## Slide 2
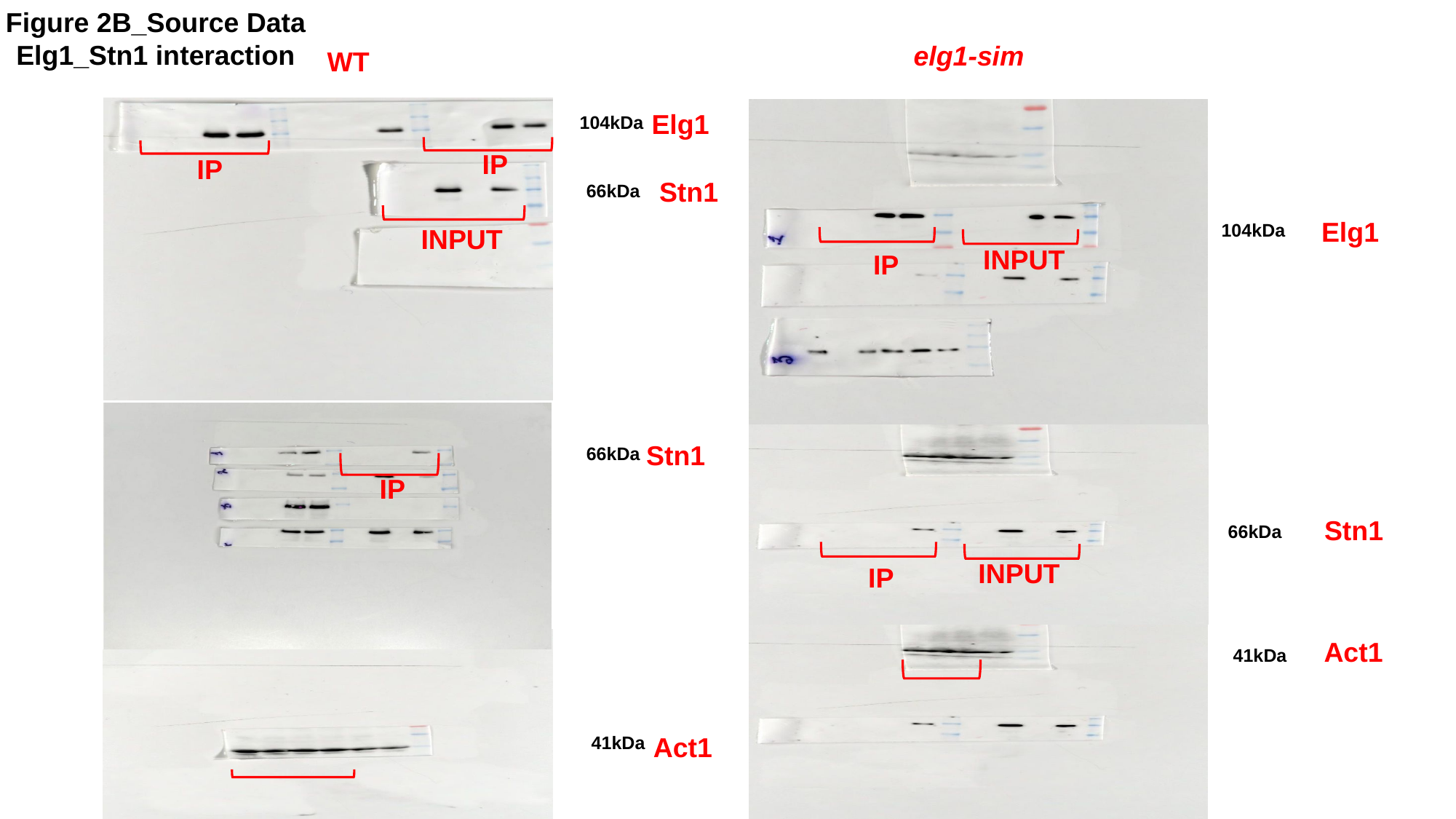

Figure 2B_Source Data
Elg1_Stn1 interaction
elg1-sim
WT
Elg1
104kDa
IP
IP
Stn1
66kDa
Elg1
104kDa
INPUT
INPUT
IP
Stn1
66kDa
IP
Stn1
66kDa
INPUT
IP
Act1
41kDa
Act1
41kDa

## Slide 3
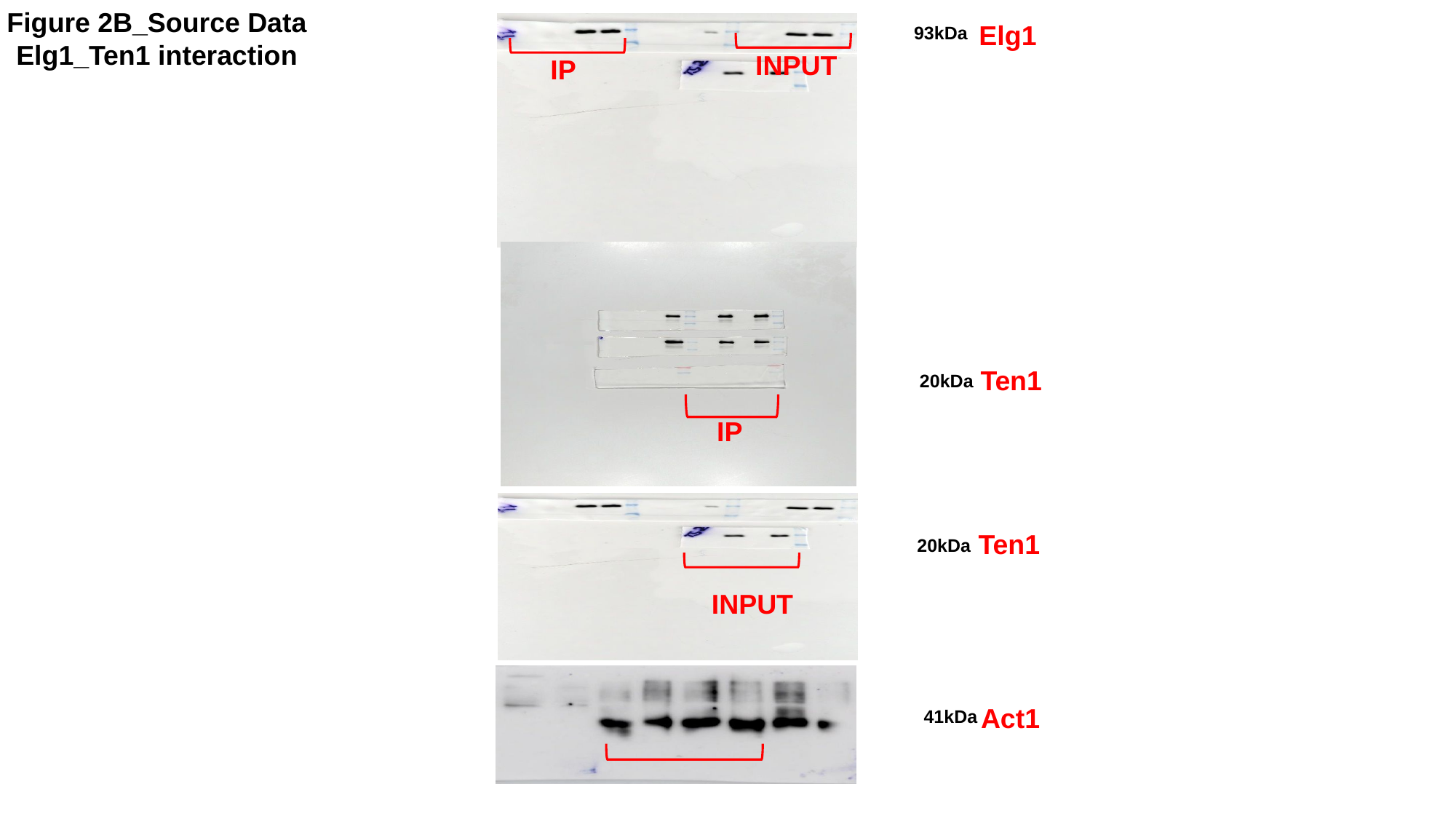

Figure 2B_Source Data
Elg1_Ten1 interaction
Elg1
93kDa
INPUT
IP
Ten1
20kDa
IP
Ten1
20kDa
INPUT
Act1
41kDa
