# Supplementary figures and images for: Control of telomere length in yeast by SUMOylated PCNA and the Elg1 PCNA unloader
Source: eLife. 2023 Aug 2;12:RP86990. doi: 10.7554/eLife.86990 (PMC10396338; doi:10.7554/eLife.86990)

## Slide 1
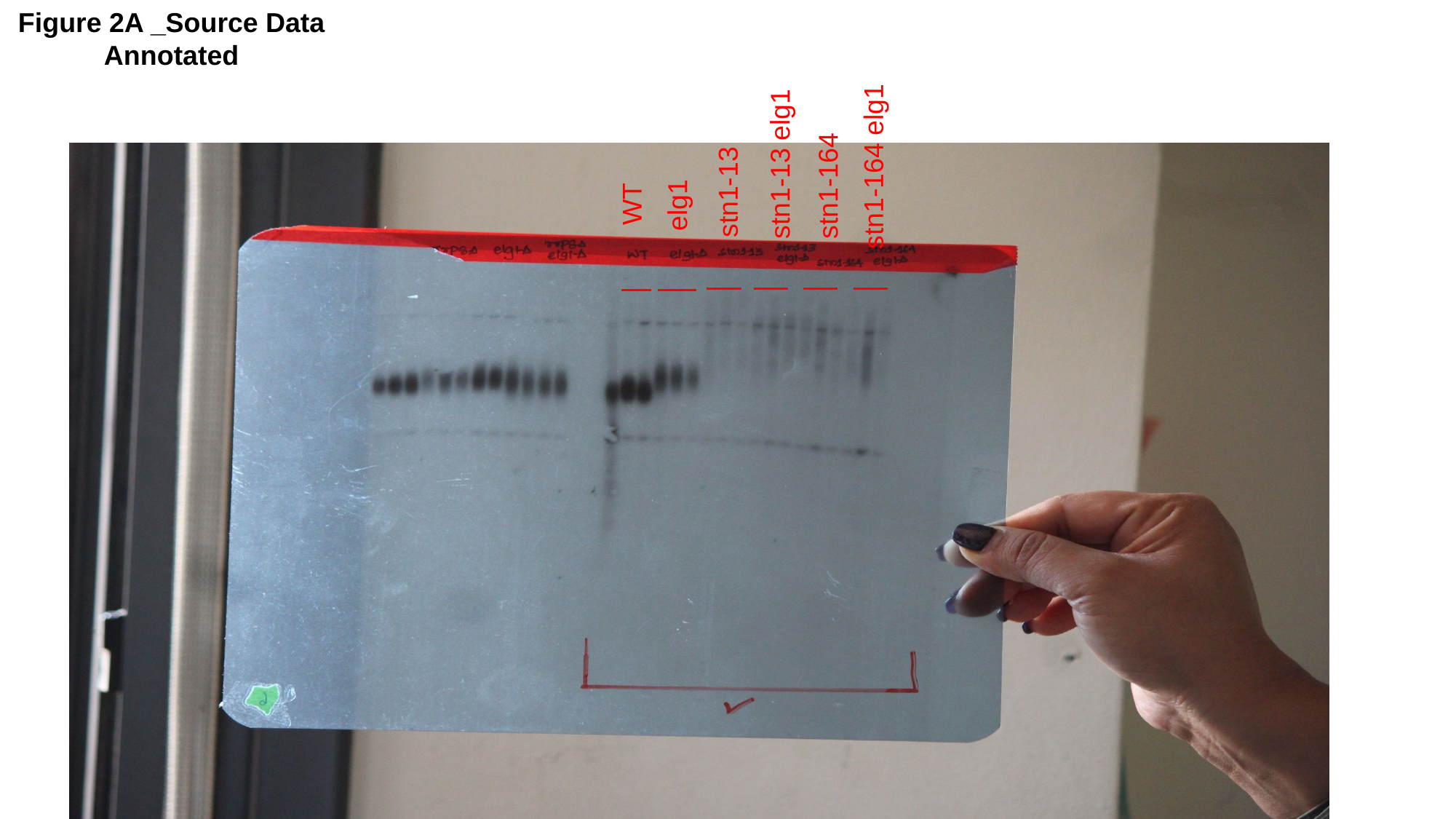

Figure 2A _Source Data Annotated
stn1-13 elg1
stn1-164 elg1
stn1-164
stn1-13
WT
elg1

Supplement: Figure 2—source data 1. [file elife-86990-fig2-data1.zip › Figure 2/Fig 2A annotated.pptx]

## Slide 1
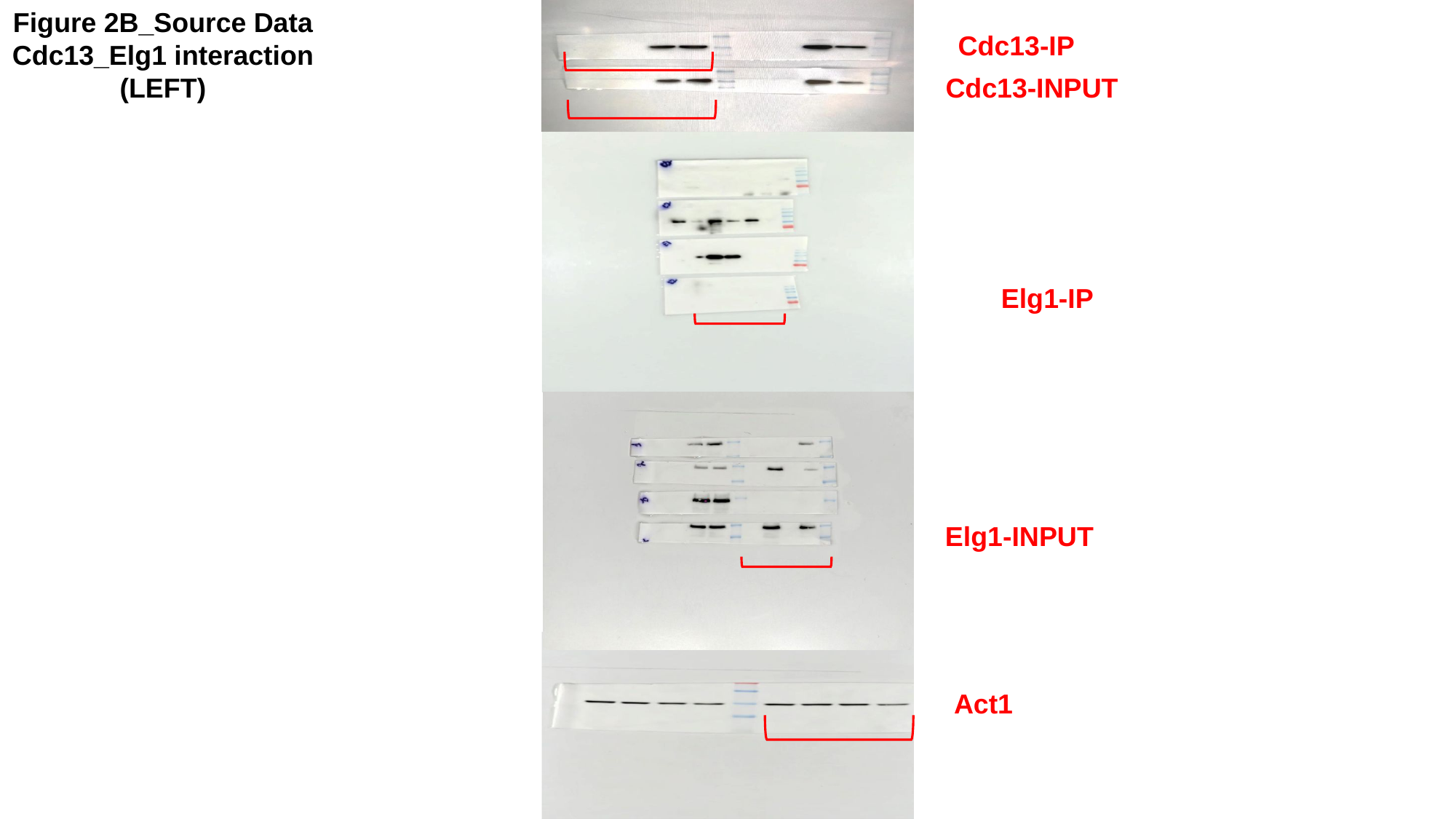

Figure 2B_Source Data
Cdc13_Elg1 interaction (LEFT)
Cdc13-IP
Cdc13-INPUT
Elg1-IP
Elg1-INPUT
Act1

Supplement: Figure 2—source data 1. [file elife-86990-fig2-data1.zip › Figure 2/Fig 2BLeft.pptx]

## Slide 1
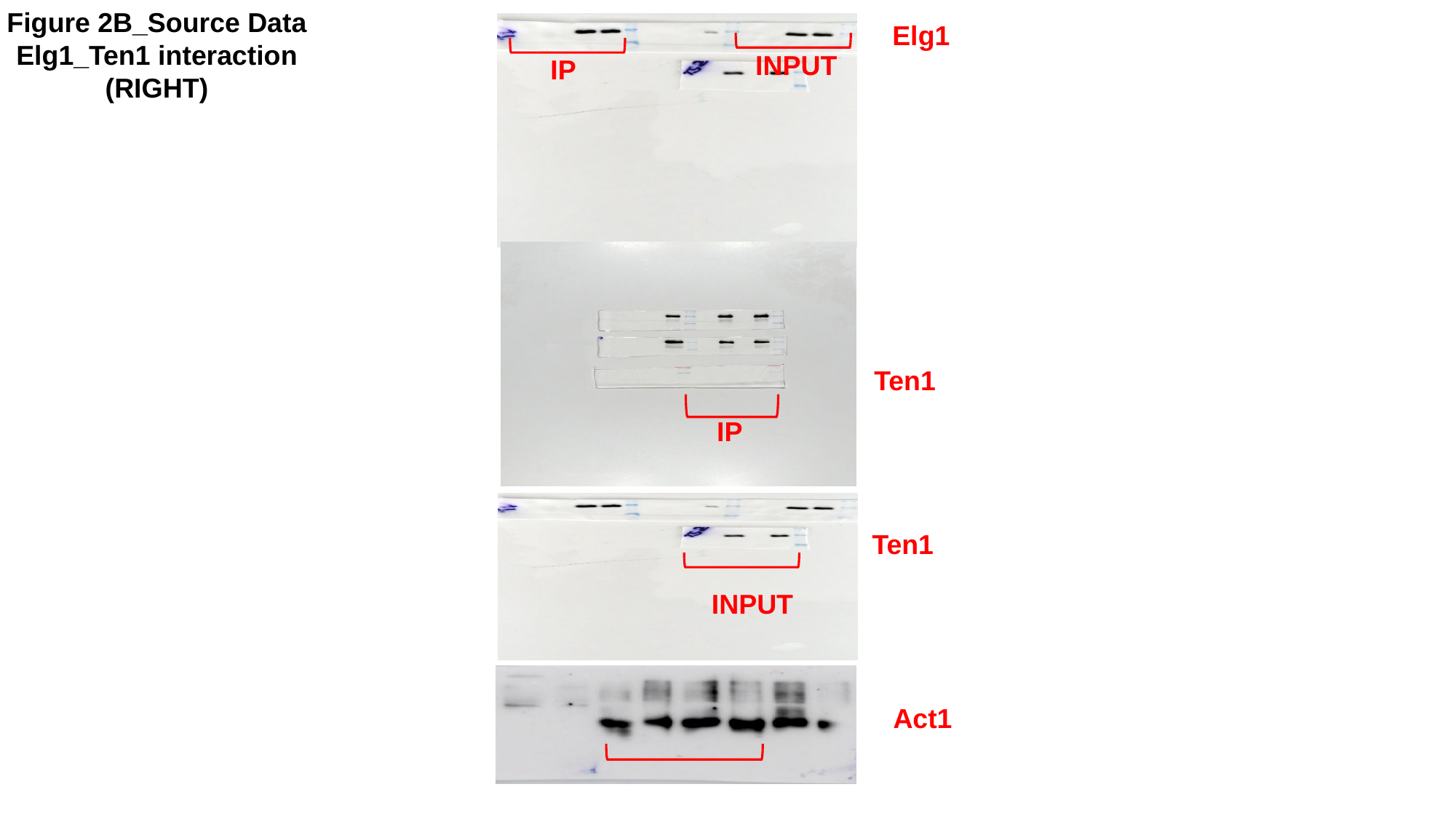

Figure 2B_Source Data
Elg1_Ten1 interaction
(RIGHT)
Elg1
INPUT
IP
Ten1
IP
Ten1
INPUT
Act1

Supplement: Figure 2—source data 1. [file elife-86990-fig2-data1.zip › Figure 2/Fig2BRIGHT.pptx]

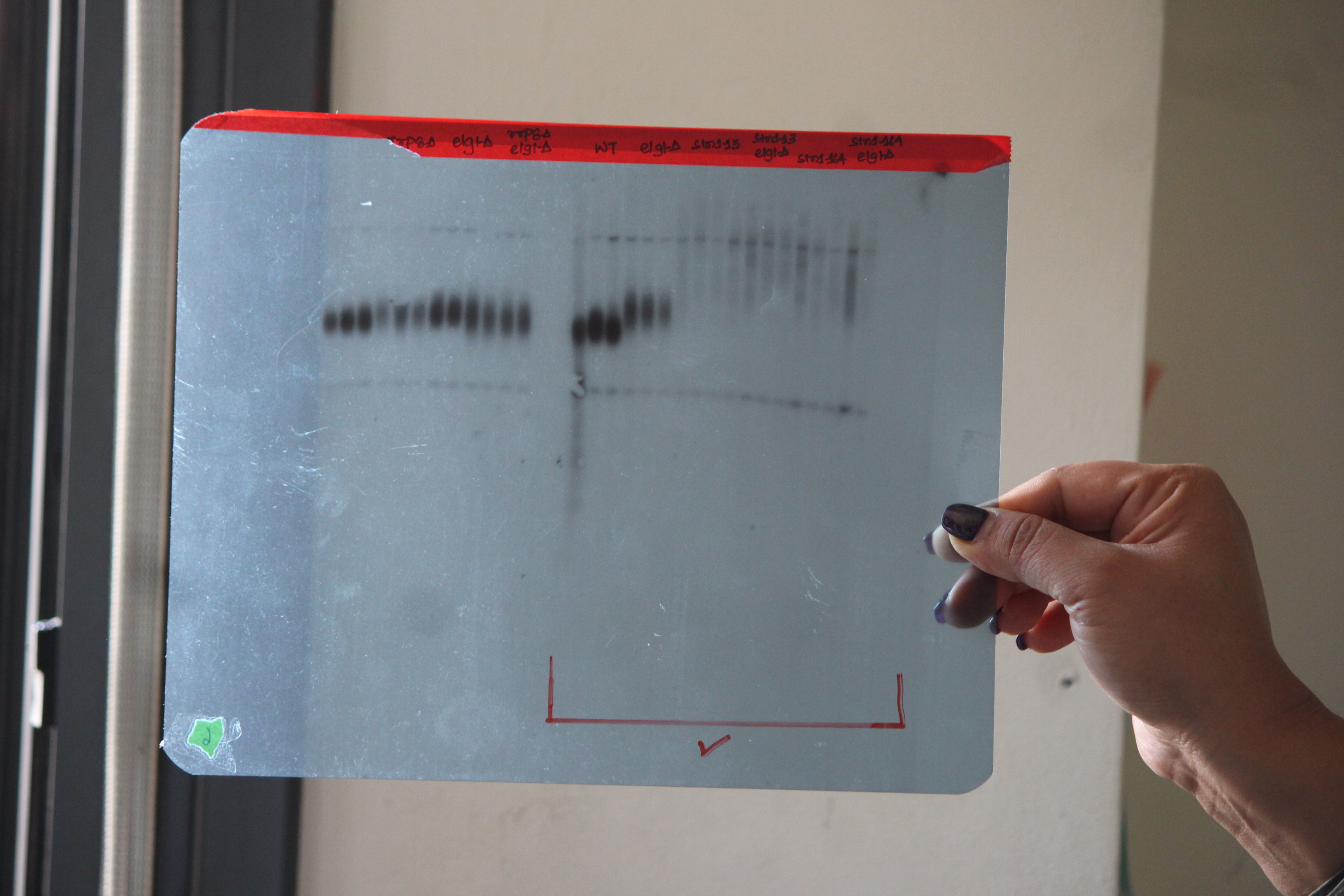

Supplement: Figure 2—source data 1. [file elife-86990-fig2-data1.zip › Figure 2/Figure 2A_Source Data.JPG]

## Slide 1
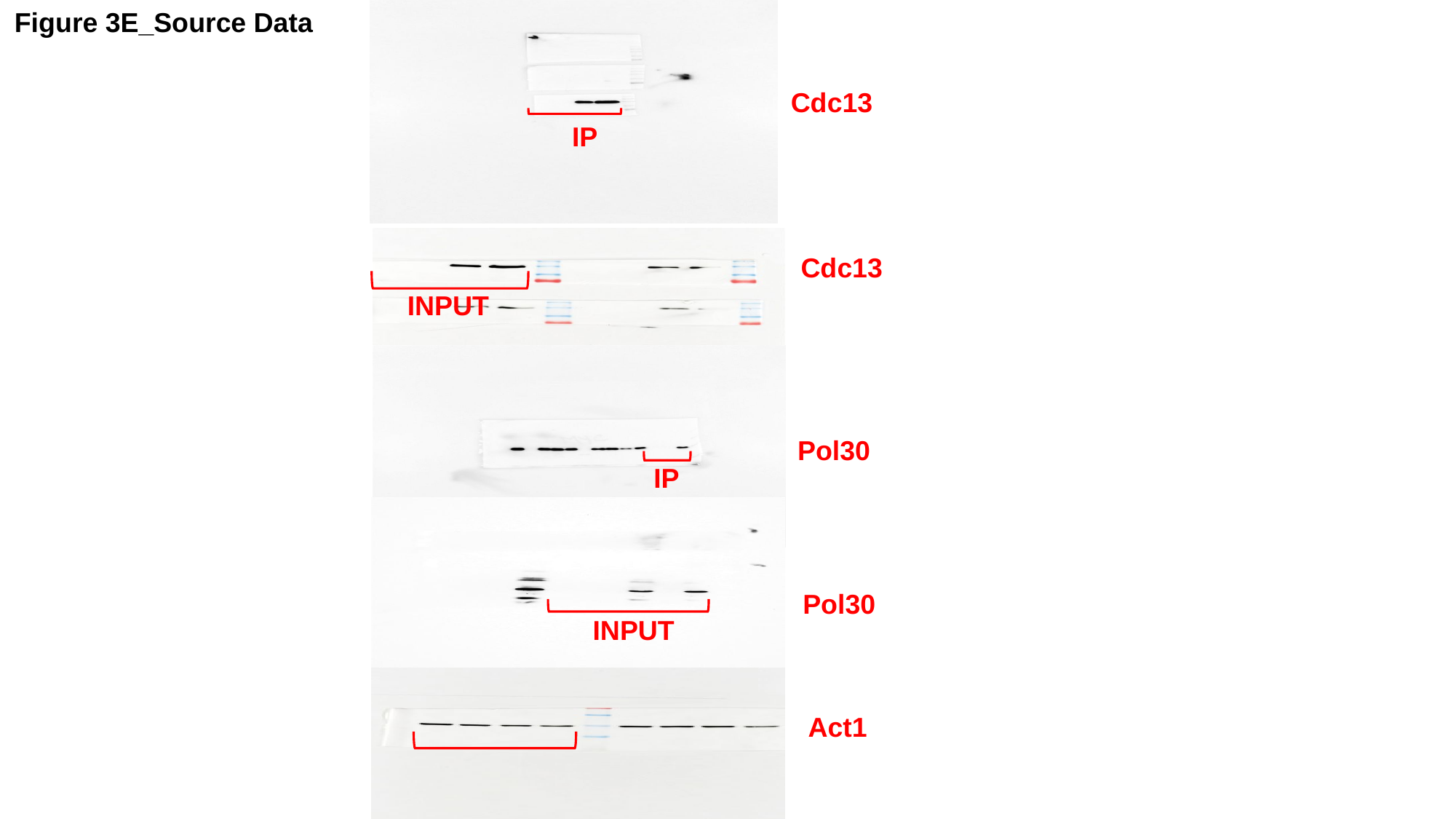

Figure 3E_Source Data
Cdc13
IP
Cdc13
INPUT
Pol30
IP
Pol30
INPUT
Act1

Supplement: Figure 3—source data 1. [file elife-86990-fig3-data1.zip › Figure 3E annotated.pptx]

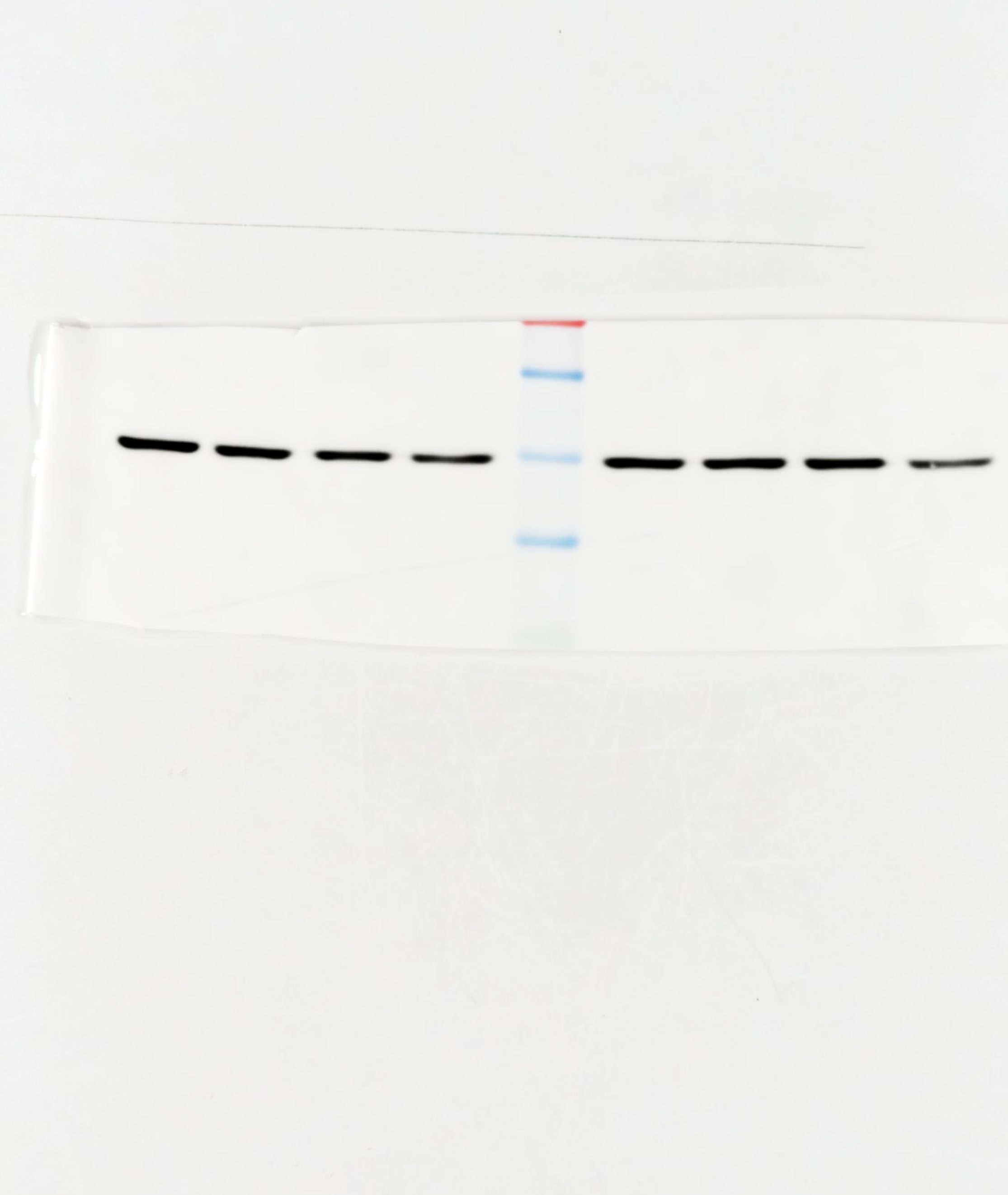

Supplement: Figure 3—source data 1. [file elife-86990-fig3-data1.zip › Figure 3E_Actin.jpg]

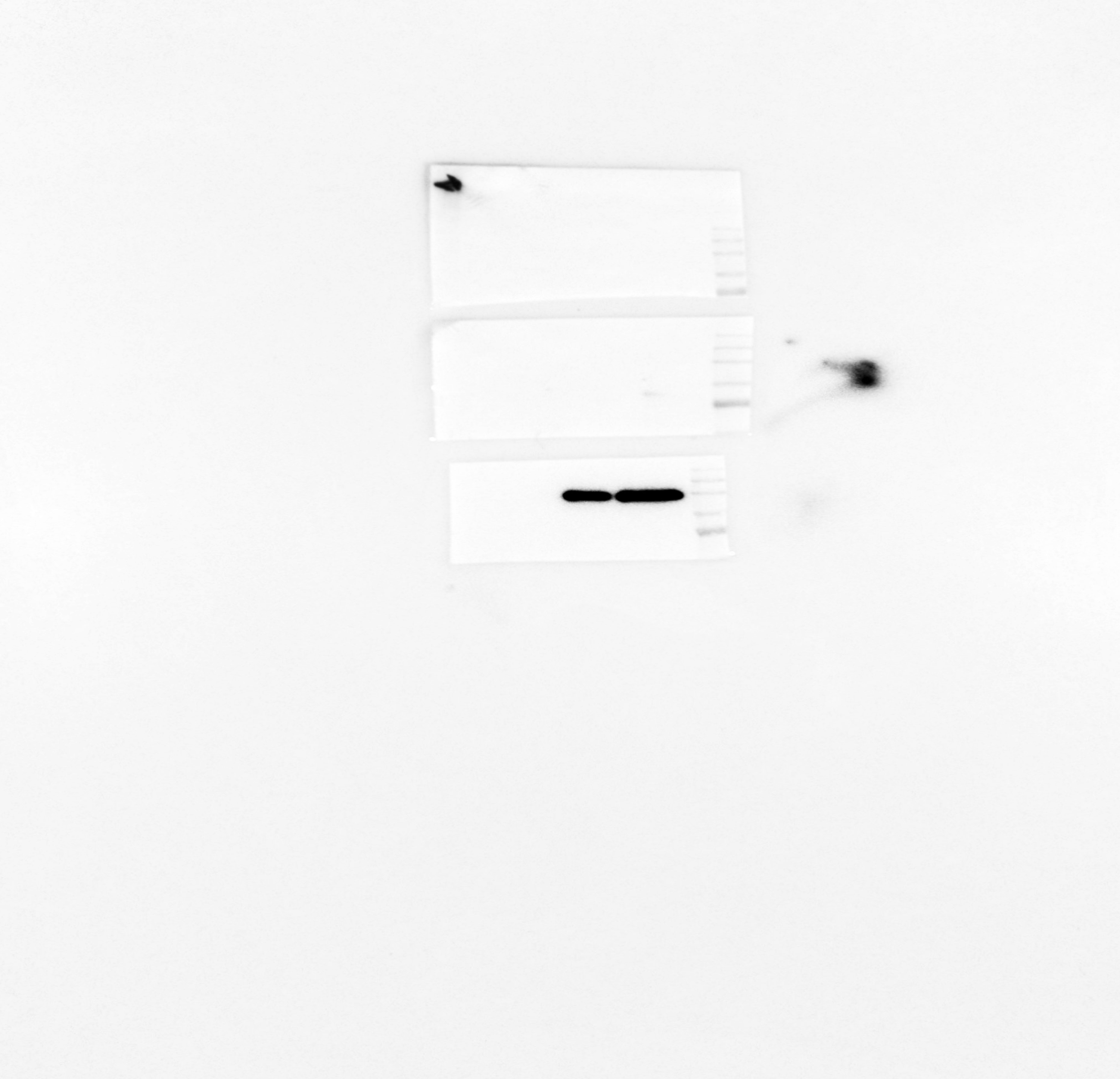

Supplement: Figure 3—source data 1. [file elife-86990-fig3-data1.zip › Figure 3E_Cdc13_IP.jpg]

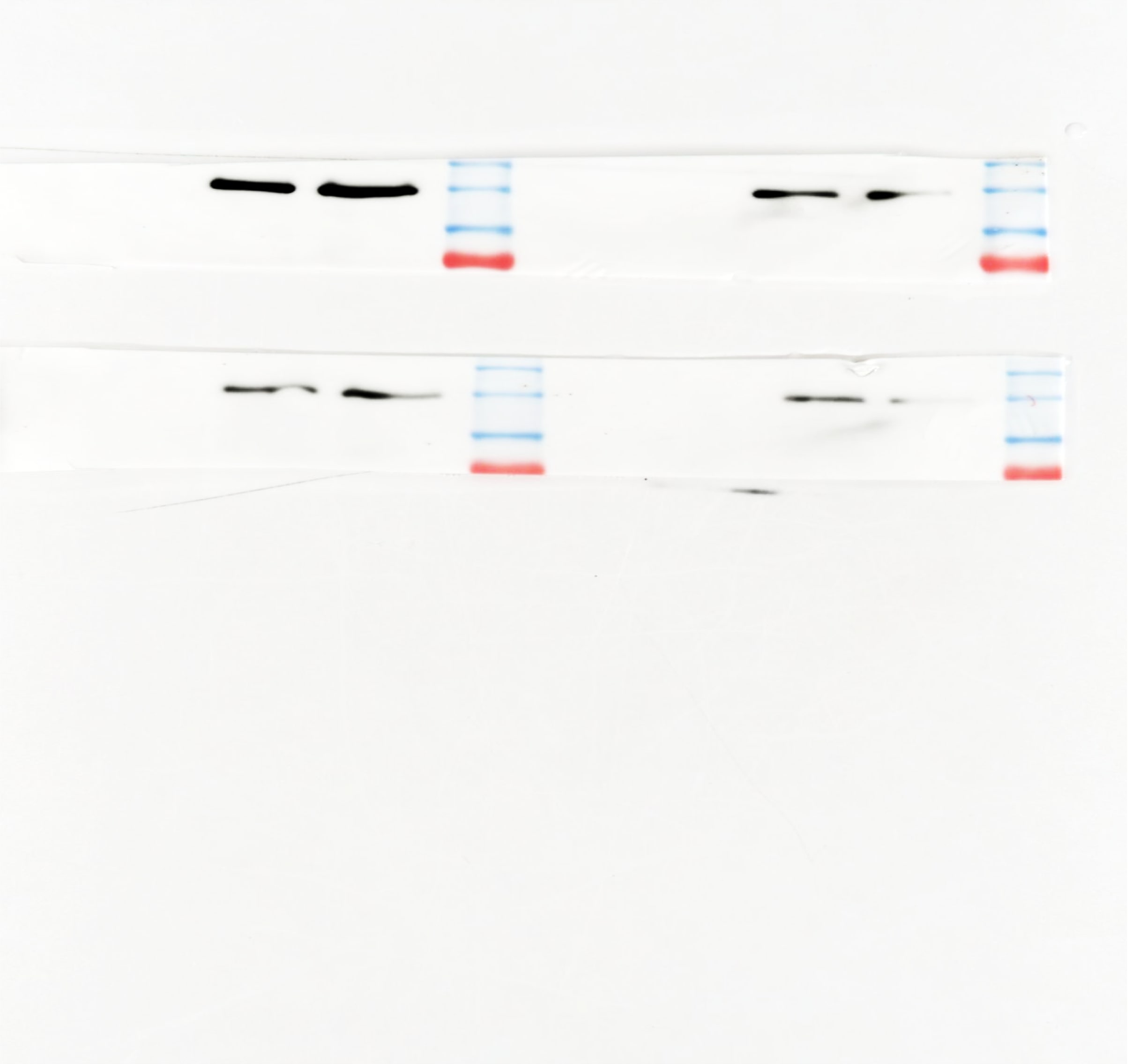

Supplement: Figure 3—source data 1. [file elife-86990-fig3-data1.zip › Figure 3E_Cdc13_WCE.jpg]

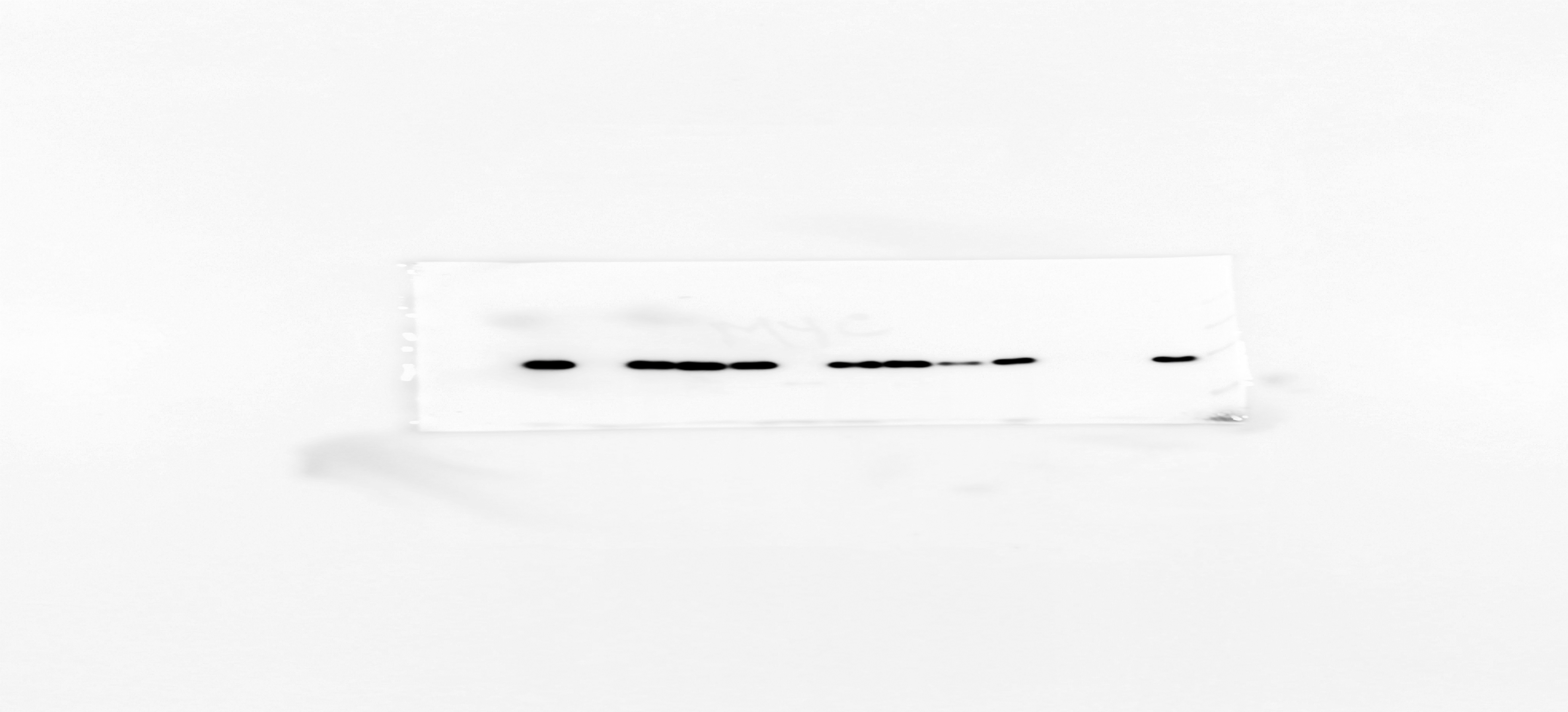

Supplement: Figure 3—source data 1. [file elife-86990-fig3-data1.zip › Figure 3E_Pol30_IP.jpg]

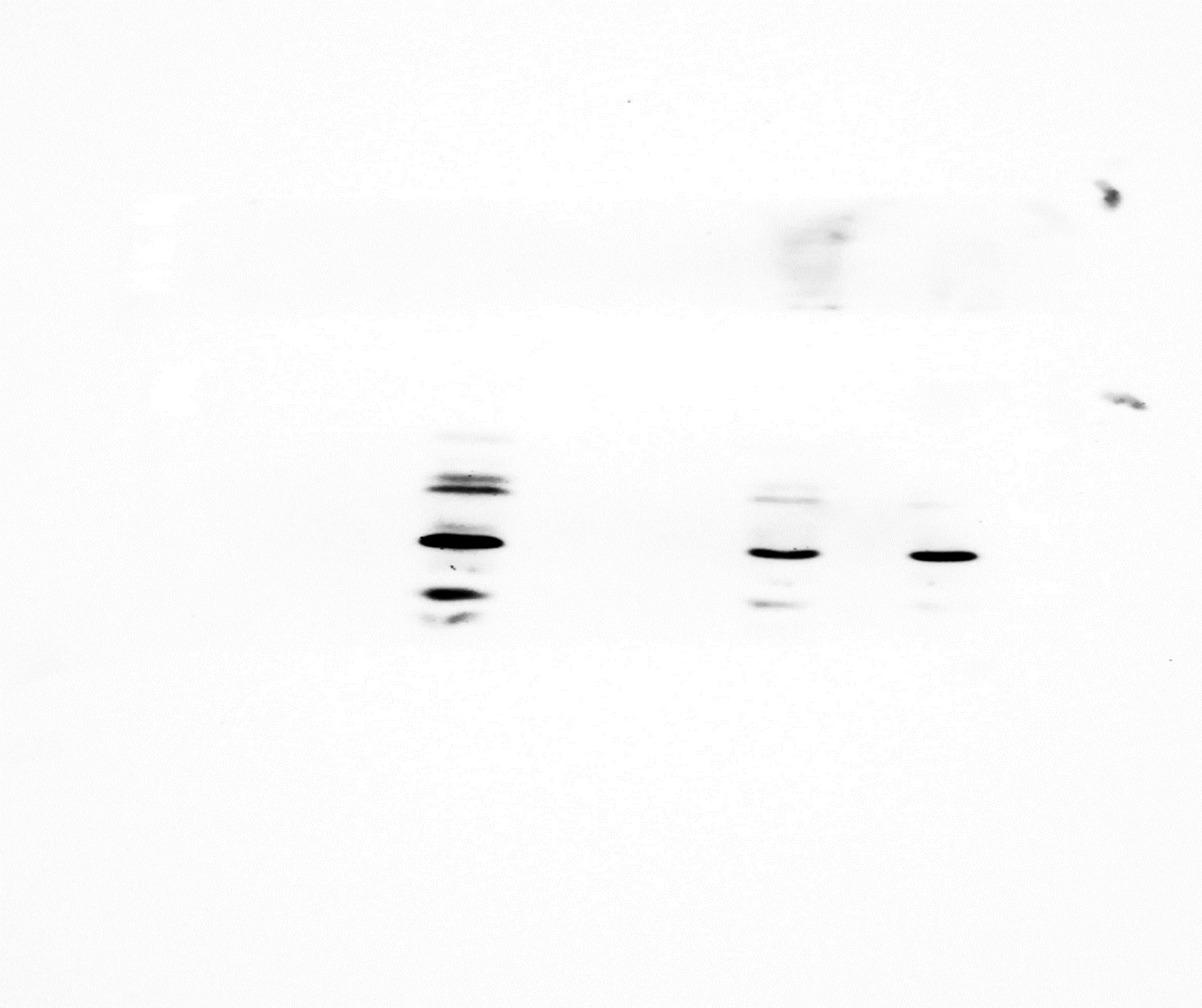

Supplement: Figure 3—source data 1. [file elife-86990-fig3-data1.zip › Figure 3E_Pol30_WCE.jpg]

## Slide 1
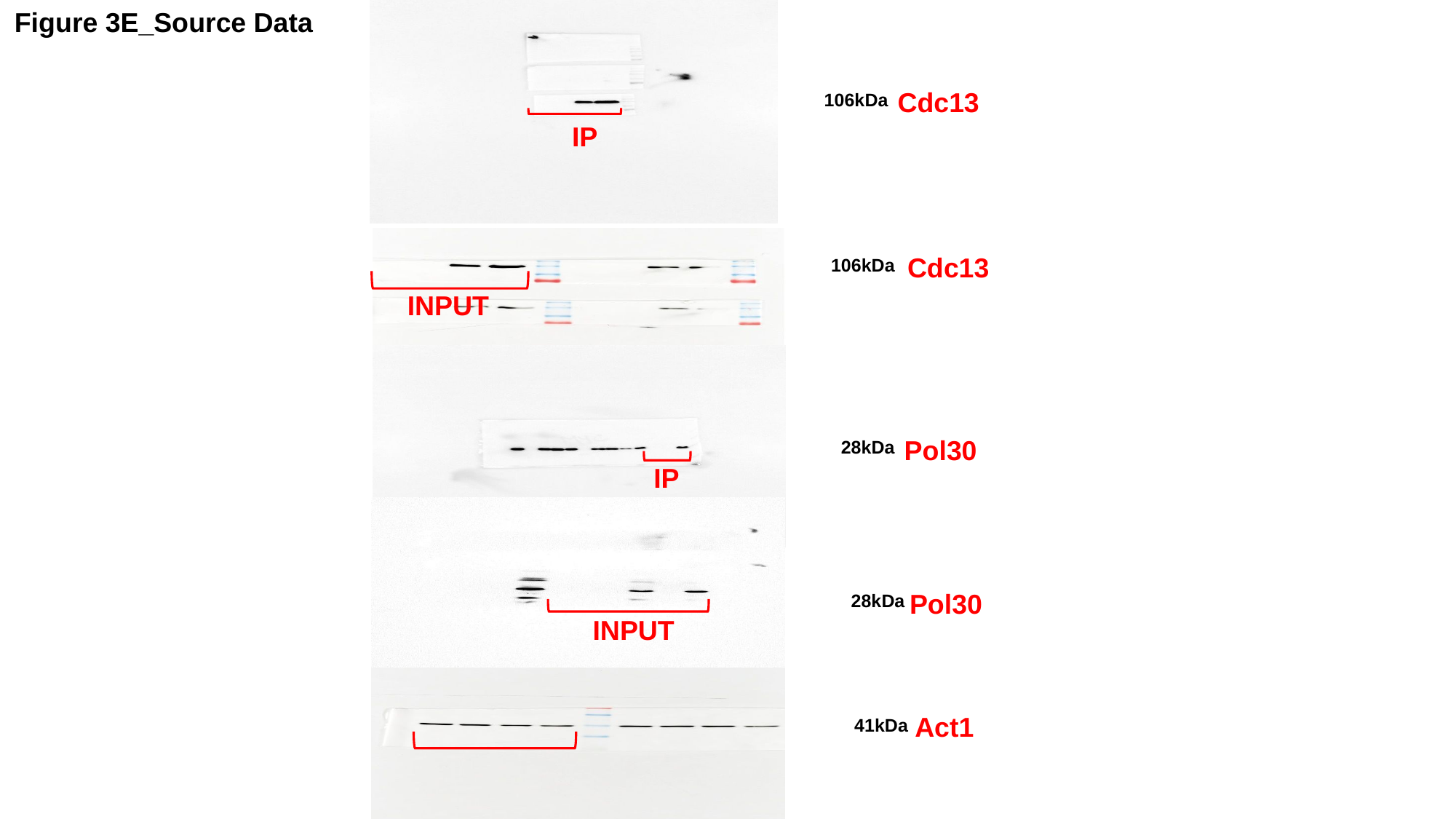

Figure 3E_Source Data
Cdc13
106kDa
IP
Cdc13
106kDa
INPUT
Pol30
28kDa
IP
Pol30
28kDa
INPUT
Act1
41kDa

Supplement: Figure 3—source data 1. [file elife-86990-fig3-data1.zip › Figure 3E with MWM.pptx]

## Slide 1
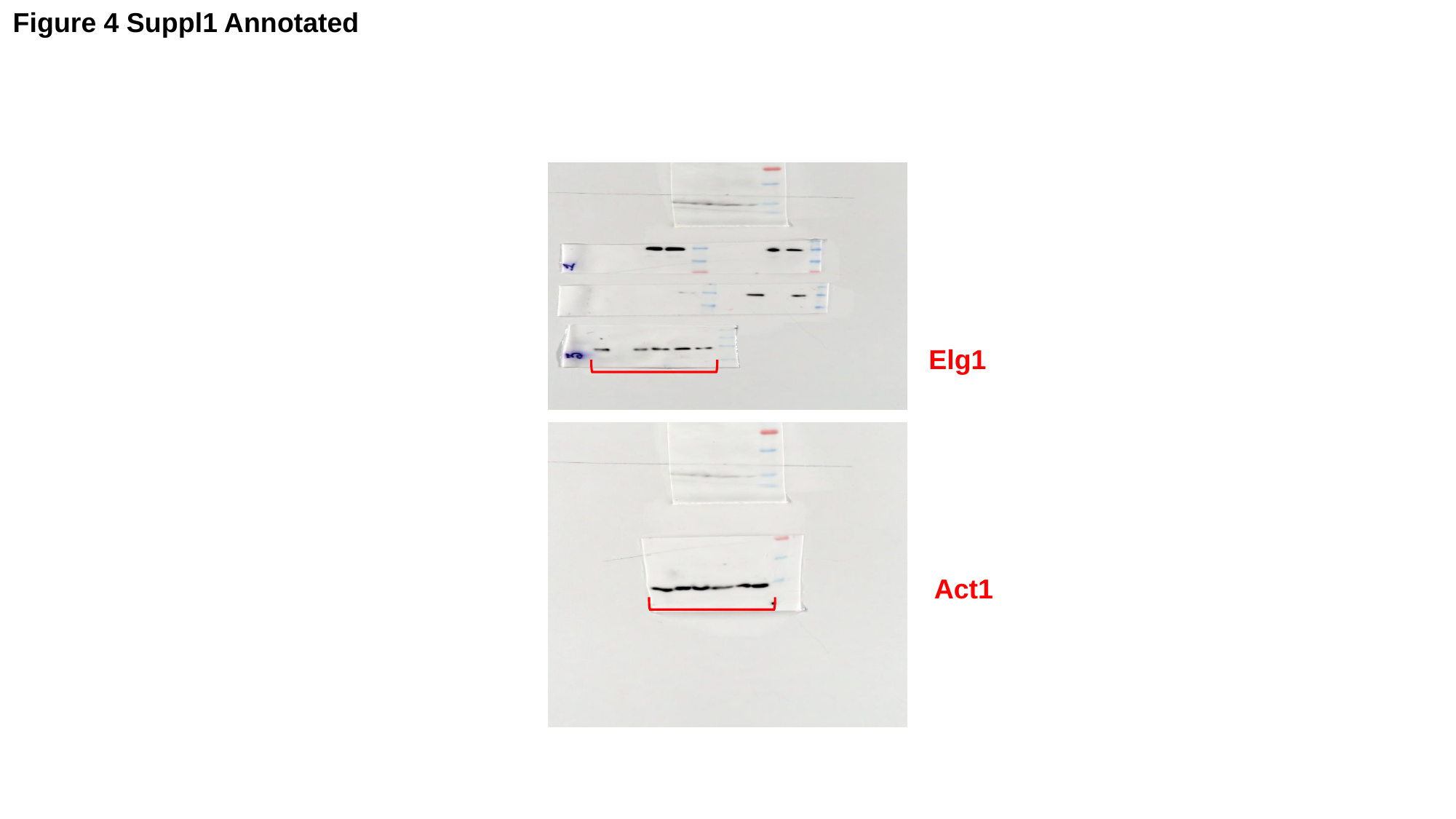

Figure 4 Suppl1 Annotated
Elg1
Act1

Supplement: Figure 4—figure supplement 1—source data 1. [file elife-86990-fig4-figsupp1-data1.zip › Figure4-Suppl1/Fig4_Suppl1 annotated.pptx]

## Slide 1
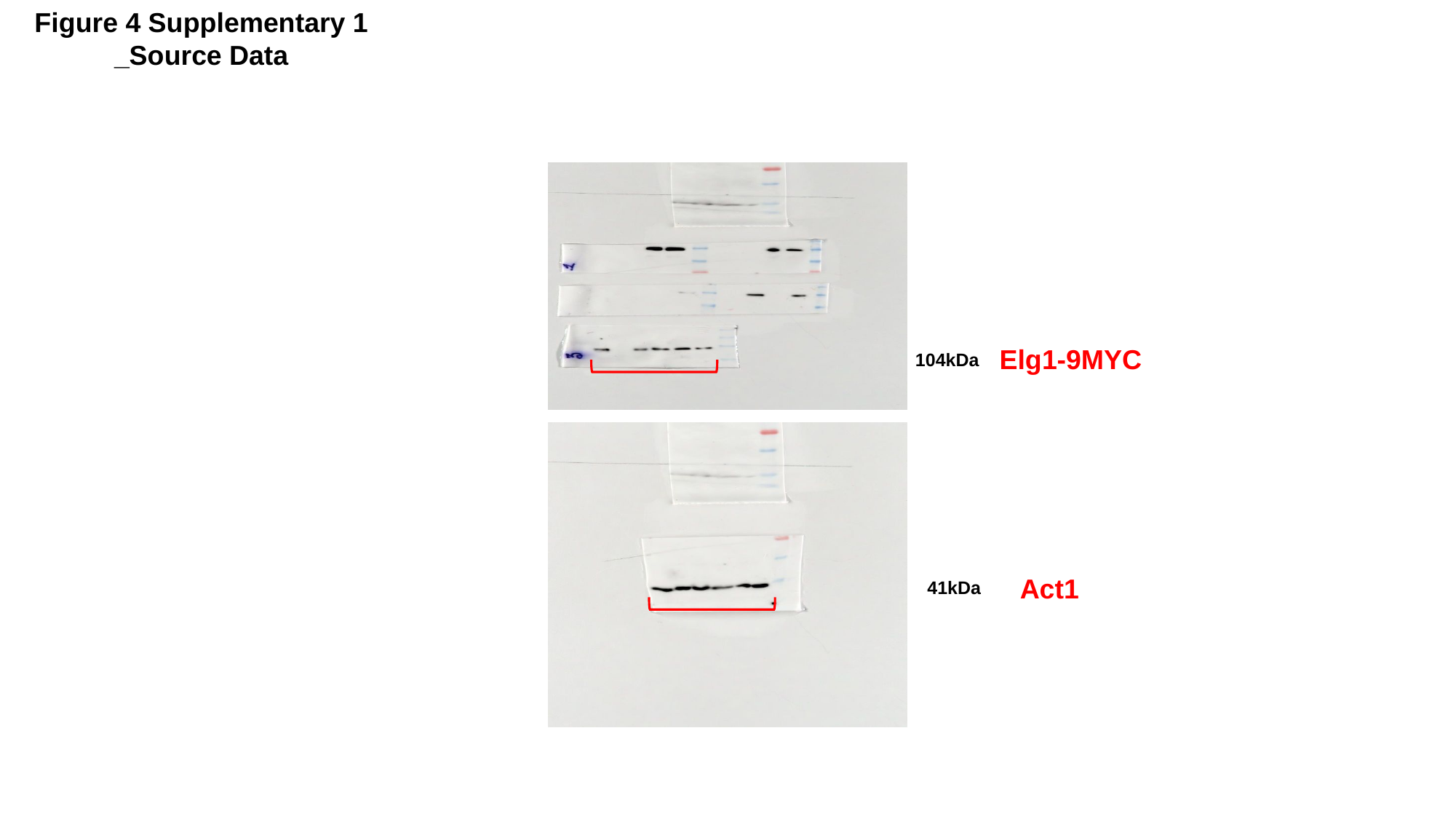

Figure 4 Supplementary 1 _Source Data
Elg1-9MYC
104kDa
Act1
41kDa

Supplement: Figure 4—figure supplement 1—source data 1. [file elife-86990-fig4-figsupp1-data1.zip › Figure 4 figure supplement 1 with MWM.pptx]

## Slide 1
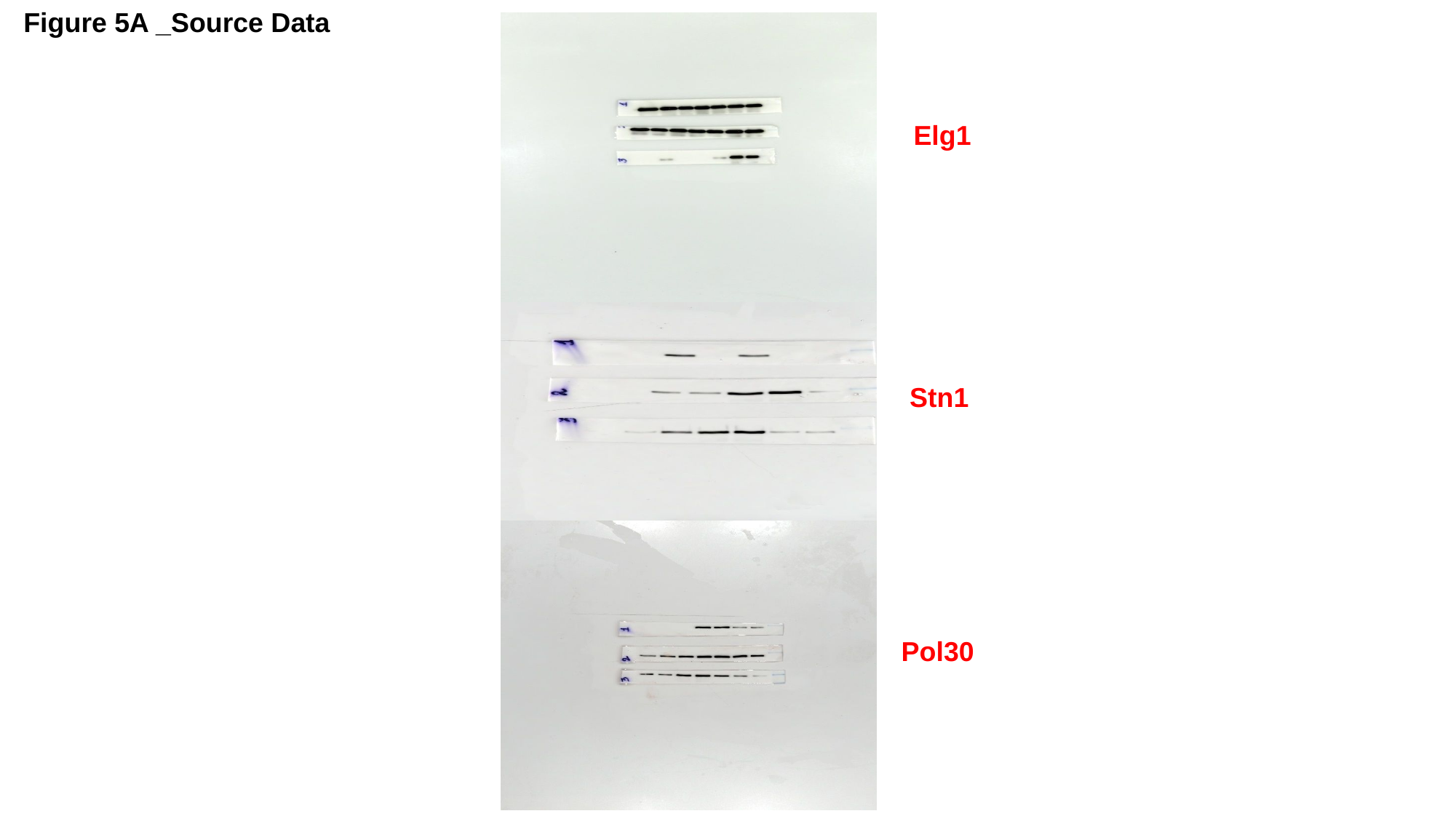

Figure 5A _Source Data
Elg1
Stn1
Pol30

Supplement: Figure 5—source data 1. [file elife-86990-fig5-data1.zip › Figure 5/Fig 5A annotated.pptx]

## Slide 1
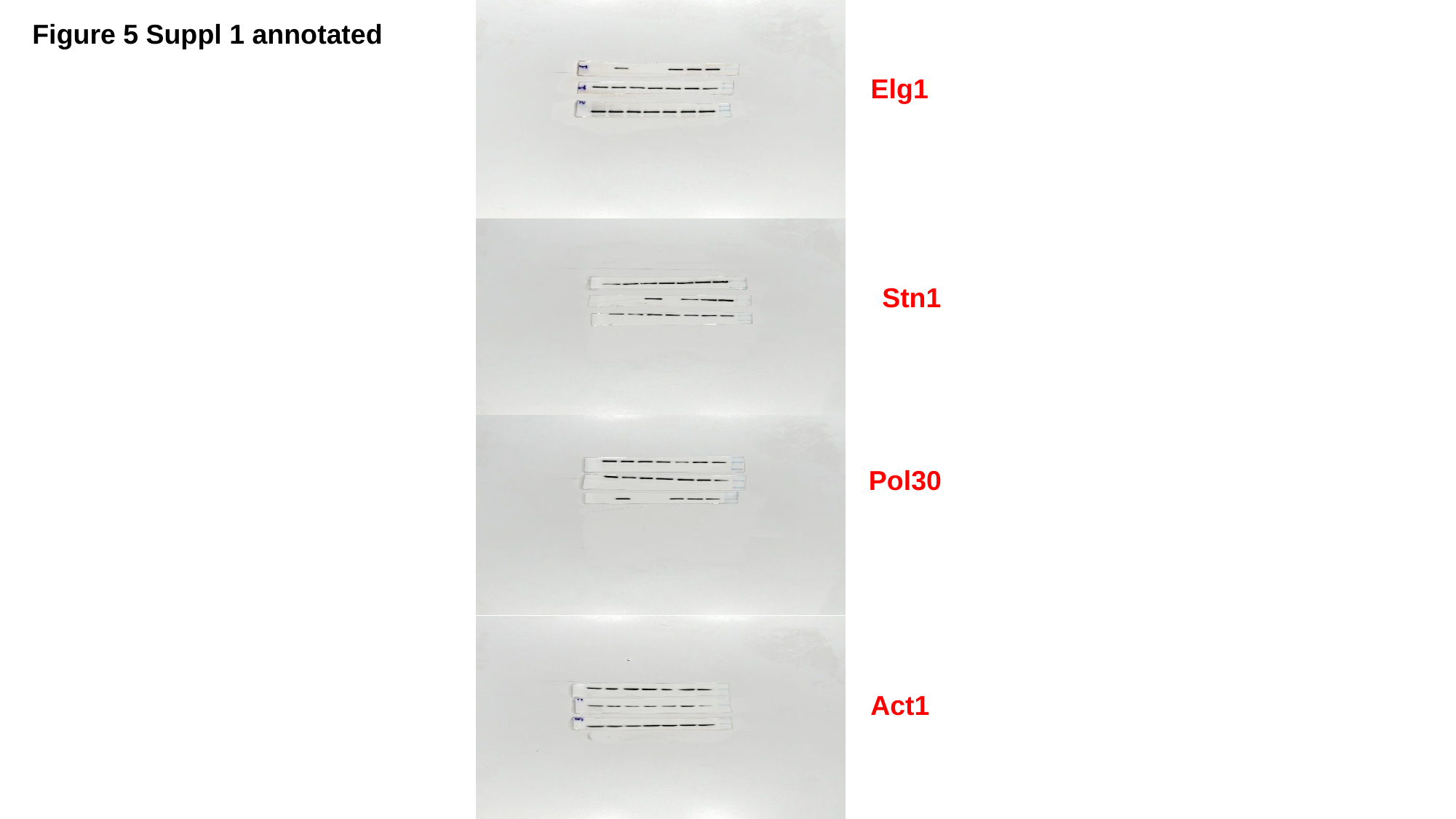

Figure 5 Suppl 1 annotated
Elg1
Stn1
Pol30
Act1

Supplement: Figure 5—figure supplement 1—source data 1. [file elife-86990-fig5-figsupp1-data1.zip › Figure 5 Suppl1/Fig 5 Suppl1 annotated.pptx]

## Slide 1
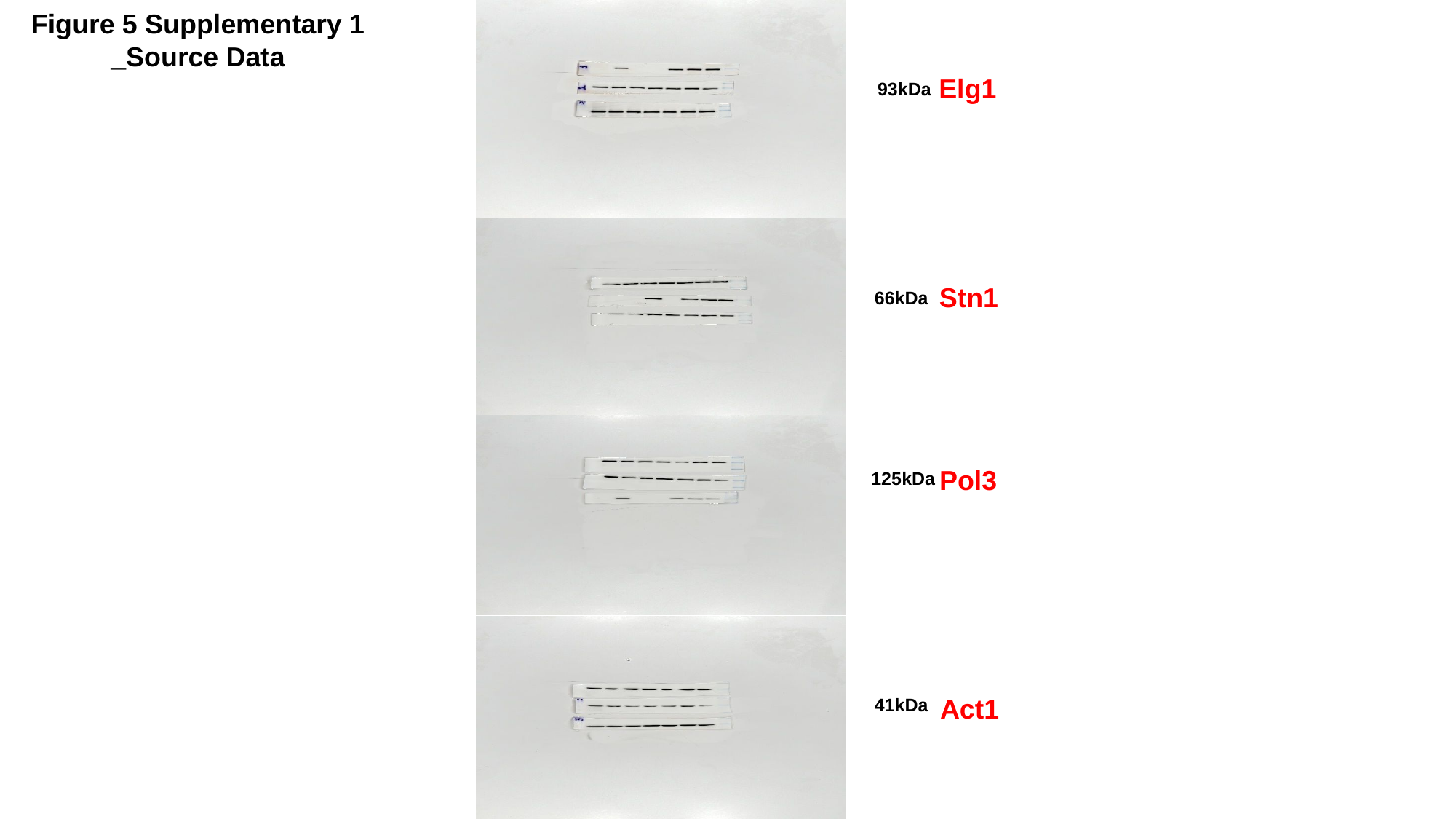

Figure 5 Supplementary 1 _Source Data
Elg1
93kDa
Stn1
66kDa
Pol3
125kDa
Act1
41kDa

Supplement: Figure 5—figure supplement 1—source data 1. [file elife-86990-fig5-figsupp1-data1.zip › Figure 5 figure supplement 1 with MWM.pptx]
