# Supplementary material for: Control of telomere length in yeast by SUMOylated PCNA and the Elg1 PCNA unloader
Source: eLife. 2023 Aug 2;12:RP86990. doi: 10.7554/eLife.86990 (PMC10396338; doi:10.7554/eLife.86990)
Supplement: Figure 5—source data 1. [file elife-86990-fig5-data1.zip › Figure 5 with MWM.pptx]

## Slide 1
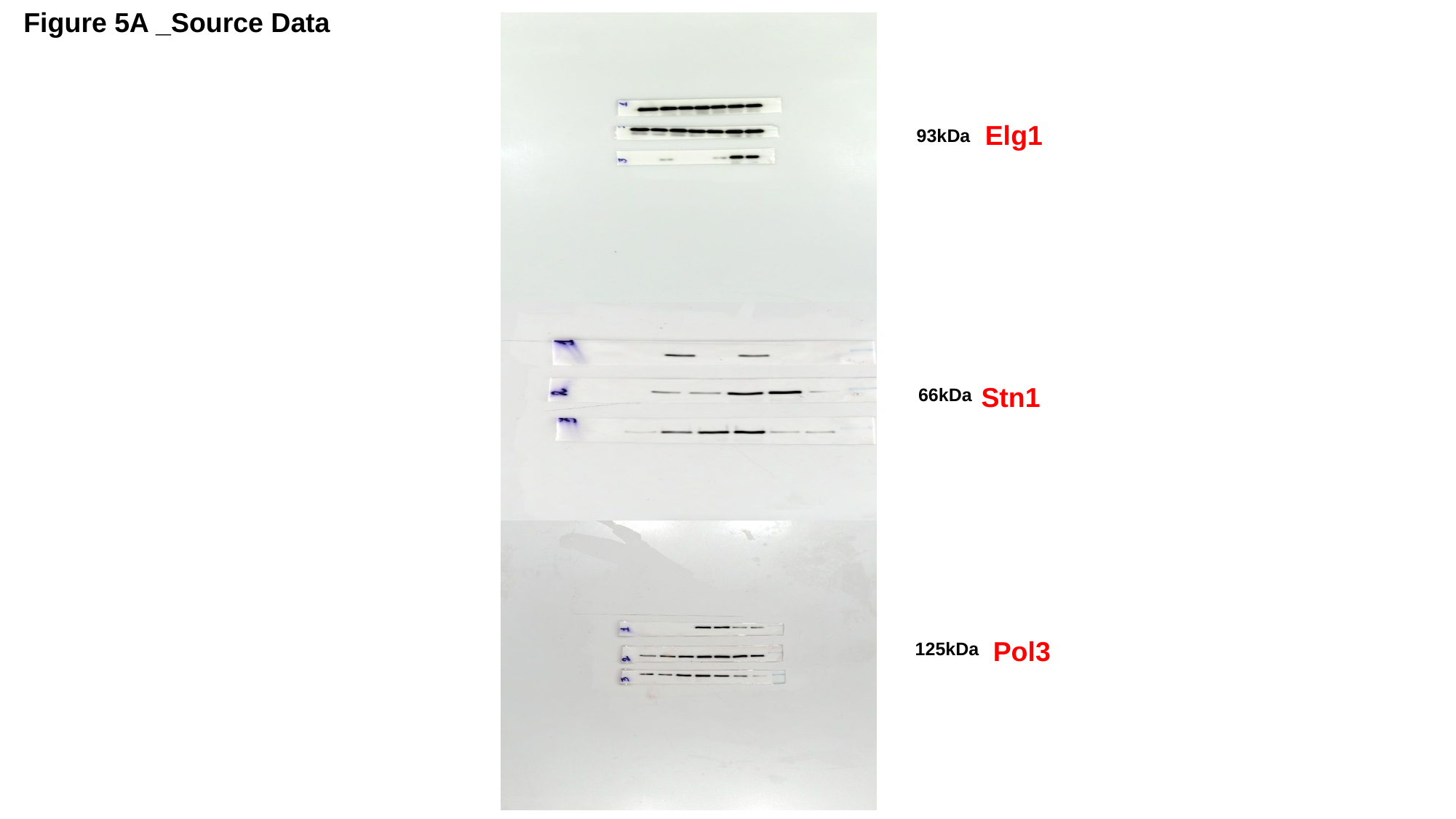

Figure 5A _Source Data
Elg1
93kDa
Stn1
66kDa
Pol3
125kDa

## Slide 2
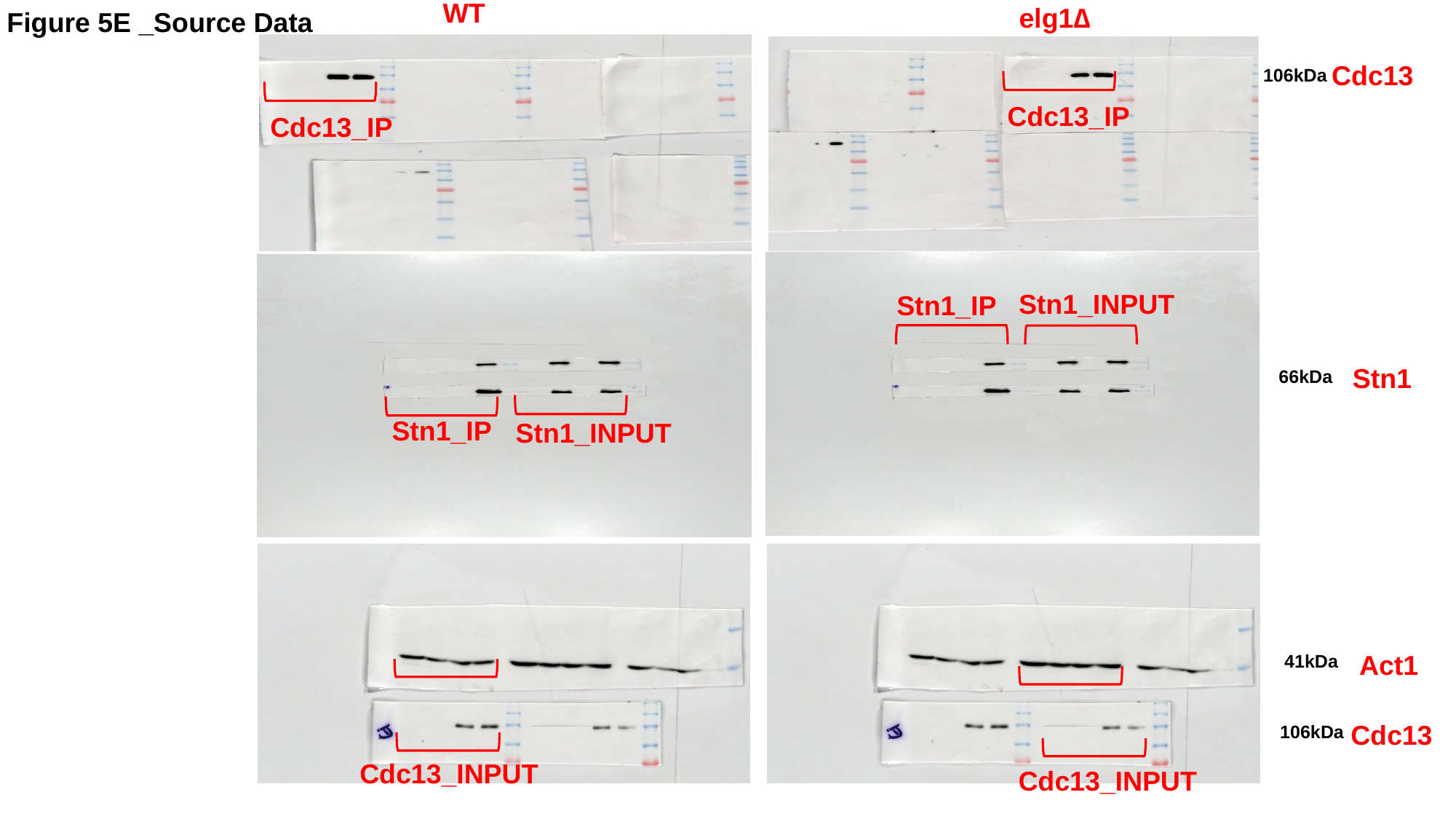

WT
elg1∆
Figure 5E _Source Data
Cdc13
106kDa
Cdc13_IP
Cdc13_IP
Stn1_INPUT
Stn1_IP
Stn1
66kDa
Stn1_IP
Stn1_INPUT
Act1
41kDa
Cdc13
106kDa
Cdc13_INPUT
Cdc13_INPUT
